# Seasonal fluctuation of beak and feather disease virus (BFDV) infection in wild Crimson Rosellas (*Platycercus elegans*)

**DOI:** 10.1038/s41598-020-64631-y

**Published:** 2020-05-12

**Authors:** Johanne M. Martens, Helena S. Stokes, Mathew L. Berg, Ken Walder, Andrew T. D. Bennett

**Affiliations:** 1 Centre for Integrative Ecology, School of Life and Environmental Sciences, Deakin University, 75 Pigdons Road, Waurn Ponds, VIC 3216 Australia; 2 Centre for Molecular and Medical Research, School of Medicine, Deakin University, 75 Pigdons Road, Waurn Ponds, VIC 3216 Australia

**Keywords:** Ecology, Zoology, Natural hazards, Diseases

## Abstract

Understanding patterns of pathogen emergence can help identify mechanisms involved in transmission dynamics. Beak and feather disease virus (BFDV) poses a major threat world-wide to wild and captive parrots. Yet data from wild birds on seasonal fluctuations in prevalence and infection intensity, and thereby the potential high-risk times for virus transmission, have been lacking. We screened wild Crimson Rosellas (*Platycercus elegans*) for BFDV in blood and cloacal swabs. Prevalence in blood samples and cloacal swabs, as well as viral load varied with Julian date and in blood, were highest after the breeding season. Breeding birds had lower viral load and lower BFDV prevalence in blood than non-breeding birds (10.1% prevalence in breeding vs. 43.2% in non-breeding birds). BFDV prevalence was much higher in younger (<3 years) than older (≥3 years) birds for both blood samples (42.9% vs. 4.5%) and cloacal swabs (56.4% vs. 12.3%). BFDV status in blood and cloacal samples was not correlated within individuals. We show that, at least in *P. elegans*, BFDV infection seems to occur year-round, with seasonal changes in prevalence and load found in our samples. Our analyses suggest that the seasonal changes were associated primarily with the breeding season. We also discuss age and sex as important predictors of BFDV infection.

## Introduction

Emerging infectious diseases of wildlife pose a major threat to the conservation of global biodiversity^1,2^. Knowledge of temporal patterns of infection allows improved targeting of management strategies to times when pathogens are most prevalent^3,4^. Pathogens are often present in host populations year-round, but prevalence fluctuates seasonally^5^. Several mechanisms have been proposed to explain this, including seasonal changes in weather^3,6^, in pathogen virulence and presence^5^, and variation in host susceptibility^7,8^. Seasonal breeding can influence host susceptibility, with lowered antibody production and increased parasite load during the breeding season often typical^9^ and generally attributed to a weakened immune system during offspring care^3^. Only a few wildlife studies have investigated the influence of seasonal changes in host biology on pathogen dynamics, with the available studies finding a peak in pathogen prevalence after the breeding season, and explaining this via an influx of susceptible young into the host population^6,10^.

Among pathogens, viruses have a particularly severe impact on wildlife, as they can adapt quickly to a wide range of potential hosts and are responsible for a high proportion of emerging infectious diseases^11^. Beak and feather disease virus (BFDV) is an ssDNA circovirus with a global distribution that can infect most, if not all, Psittaciformes (parrots, lorikeets, cockatoos)^12^. This is concerning, as the Psittaciformes are among the most threatened bird orders^13^. Accordingly, BFDV has been declared a ‘key threatening process to biodiversity’ by the Australian Government and a Threat Abatement Plan has been established^14,15^. It has furthermore been found in an increasing number of non-psittacine birds^16–18^. BFDV can be transmitted horizontally through crop secretions, faeces and feather dander^19^, and vertically from mother to embryonated egg^20^. It can cause acute and mostly fatal disease in very young birds, as well as chronic, often fatal disease mostly in older individuals^21^, and has been shown to persist for up to at least seven months in wild host individuals^22^. The risk of severe consequences of infection with BFDV is especially high in small populations of endangered species with low genetic diversity^23–25^. BFDV presence can become a particularly high-risk factor where distributions of abundant hosts overlap with those of vulnerable species^12^. Data on prevalence and spread in wild populations, even abundant ones which may act as pathogen reservoirs, are rare but urgently needed for species conservation^12^. Possible seasonal fluctuations in BFDV prevalence have been discussed^26^, but good data on seasonal prevalence and potential high-risk times are lacking. Such data are however necessary for a targeted, more effective pathogen management approach by conservationists, and could lead to a better understanding of BFDV infection dynamics.

BFDV causes disease with severe signs in many, but not all species of Psittaciformes^27^. Psittacine Beak and Feather Disease (PBFD) is the most common disease in wild Australian psittacines^28^. Severity of infection can range from subclinical, with no signs or mild signs, to severe clinical disease with feather dystrophy and abnormal beak and claw growth^27,29^. Many infected birds seem to stay asymptomatic^12^; yet asymptomatic birds play an important role as virus shedders^30^. Individuals that are infected with BFDV usually excrete large amounts of virus in feather dander and faeces^19,31^, often while giving PCR-negative results for BFDV if only one sample type is tested^32^.

Crimson Rosellas (*Platycercus elegans*) are an abundant and wide-spread Australian parrot which may act as a reservoir host for BFDV^33^. In the *P. elegans elegans* subspecies, BFDV prevalence of 34.5% has been reported^34^. *P. elegans* show no to only mild signs of BFDV infection, which can be challenging to detect in observation-based disease monitoring^27^. It has been proposed that BFDV prevalence in wild *P. elegans* might be influenced by time of year or season^33^, but studies with systematic sampling throughout the year to investigate this have been lacking. Such screening can provide a foundation for targeted pathogen management plans and for studies on host susceptibility^12^. A previous study showed that within breeding pairs of *P. elegans*, BFDV prevalence was lower than expected by random mating, and positive assortative mating of individuals without BFDV infection was suggested as a possible mechanism^35^. However, no study has investigated the patterns of BFDV prevalence between breeding and non-breeding birds, for a better understanding of BFDV prevalence dynamics throughout the year.

We investigated the seasonality of BFDV in breeding (trapped as parental birds in nest boxes) and non-breeding (trapped outside the breeding season) wild *P. e. elegans*. We tested whether host characteristics including breeding status, age and sex were associated with prevalence and viral load (intensity of infection). We also investigated whether host body mass was related to infection status and viral load, with the aim of elucidating fitness consequences of BFDV infection^8^. We predicted that prevalence and intensity of infection would be highest in autumn (after the breeding season) due to the influx of susceptible youngsters^6,10^. Our key aims were to 1) test for seasonal variation in BFDV prevalence and viral load, as well as body mass, to identify potential high-risk times for virus transmission, and 2) test whether host breeding status and age are related to any observed seasonal changes in BFDV prevalence and load.

## Results

We collected blood samples from 142 individual *P. e. elegans* over two years (October 2016 to September 2018) including two breeding seasons (October to January). Of the 123 *P. elegans* with known breeding status, breeding birds included 36.7% (29 of 79) young birds (<3 years) and 63.3% (50 of 79) older birds (≥3 years), whereas non-breeding birds included 61.4% (27 of 44) young birds and 38.6% (17 of 44) older birds.

### BFDV prevalence in blood

**Figure 1:**
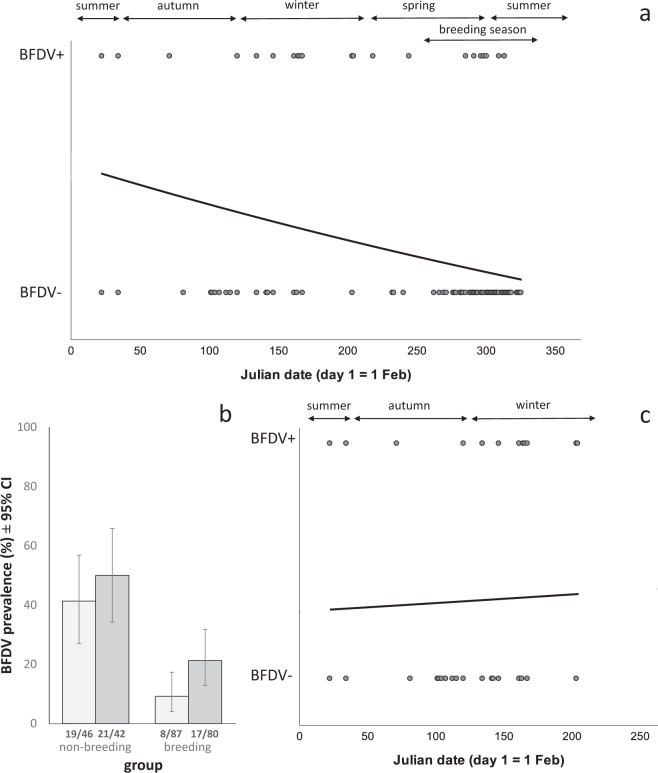
BFDV prevalence versus (**a**,**c**) date and (**b**) breeding status. ‘BFDV+’ indicates BFDV-positive birds, ‘BFDV−’ indicates BFDV-negative birds. Prevalence versus date is represented as a logistic regression curve. (**a**) Prevalence_blood_ of birds caught throughout the year, including outside the breeding season (‘non-breeding’) and during (‘breeding’) the breeding season. (**b**) Mean prevalence (with 95% confidence intervals) by breeding status, where light grey bars indicate population prevalence of BFDV in blood samples, and dark grey bars indicate population prevalence of BFDV in cloacal swabs. (**c**) Prevalence_blood_ for non-breeding birds only. In (**a**,**b**) we show only prevalence_blood_ and not prevalence_cloacal_, because cloacal swabs were not subject to the same level of validation as blood samples. Date is shown as Julian date, with the 1^st^ February, which marks the end of the breeding season, set as day 1, and so the 31^st^ January is day 365. Fractions at the base of bars indicate the number of infected birds out of the number of trapped individuals. Nine birds which were caught in walk-in traps during the breeding season are not shown in (**b**,**c**) as we could not determine whether or not they were breeding.

In blood samples, we detected BFDV in all age and sex classes of *P. e. elegans*, and the overall prevalence_blood_ (refers to population prevalence of BFDV in blood samples) was 21.8% (31 of 142 individuals, 95% confidence interval (CI) 15.3 – 29.5). When we tested the complete data set (i.e. both breeding and non-breeding birds) we did not find an effect of season on prevalence_blood_ (see Supplementary Table S3 for statistics). We did, however, find significant linear and quadratic effects of date (p = 0.005 and 0.046 respectively, Supplementary Table S3), indicating fluctuations in BFDV prevalence throughout the year: prevalence_blood_ appeared to be highest after the breeding season (1^st^ February) and then declined towards the next breeding season (Fig. 1a). Host sex and age class were also consistently related to prevalence_blood_, regardless of controlling for season, date, or breeding status. Overall, females (14 of 58, 24.1%, 95% CI 13.9 – 37.2) had a higher prevalence than males (13 of 65, 20.0%, 95% CI 11.1 – 31.8). However, when accounting for season, date, or breeding status, males were more likely to be infected than females (Supplementary Table S3, Supplementary Fig. S2). Young birds (<3 years) had a significantly higher prevalence_blood_ (24 of 56, 42.9%, 95% CI 29.7 – 56.8) than older birds (≥3 years) (3 of 67, 4.5%, 95% CI 0.9 – 12.5). This was the case when controlling for season, date, or breeding status (Supplementary Table S3). Breeding birds had a lower prevalence_blood_ (8 of 79, 10.1%, 95% CI 4.5 – 19.0) than non-breeding birds (19 of 44, 43.2%, 95% CI 28.3 – 59.0; p = 0.015, Supplementary Table S3, Fig. 1b). When we analysed non-breeding birds only, we found no relationship between prevalence_blood_ and date (p > 0.35) or season (p = 0.35), but the effects of age and sex remained (Supplementary Table S3; Fig. 1c). Mean prevalence_blood_ in young females by season and breeding status is shown in Supplementary Fig. S3 and within young birds by age in months in Supplementary Fig. S4, but sample sizes did not permit further statistical analysis for these age and sex cohorts.

### BFDV prevalence in cloacal swabs

The overall prevalence_cloacal_ (refers to population prevalence of BFDV in cloacal swabs) was 33.3% (40 of 120, 95% CI 24.9 – 41.8). An individual’s BFDV status in blood was independent of its BFDV status in cloacal swabs (p = 0.20, Supplementary Table S4). Of the 51 *P. e. elegans* with BFDV prevalence data for both blood samples and cloacal swabs, and that were also BFDV positive (BFDV+) in at least one sample type, 21.0% (11 of 51, 95% CI 10.3 – 32.9) were only BFDV+ in blood (BFDV+ _blood_), 37.3% (19 of 51, 95% CI 23.0 – 50.5) were only BFDV+ in cloacal swabs (BFDV+ _cloacal_), and 41.2% (21 of 51, 95% CI 27.7 – 54.7) were BFDV+ in both sample types.

In cloacal samples, we found a cubic relationship between date and BFDV prevalence (with prevalence increasing after the breeding season, then decreasing, then increasing again throughout the year, similar to a sinusoidal curve; p = 0.017, Supplementary Table S4). When accounting for year of study, there was no significant relationship between date and BFDV prevalence (Supplementary Table S9). In the same model, prevalence_cloacal_ (refers to population prevalence of BFDV in cloacal swabs) did not differ significantly between males and females (males: 16 of 66, 24.2%, 95% CI 14.5 – 36.4; females: 23 of 54, 42.6%, 95% CI 29.2 – 56.8; p = 0.296, Supplementary Table S4), but prevalence_cloacal_ was higher in younger than in older birds (young, <3 years: 31 of 55, 56.4%, 95% CI 42.3 – 69.7; old, ≥3 years: 8 of 65, 12.3%, 95% CI 5.5 – 22.8; p < 0.001, Supplementary Table S4). Prevalence_cloacal_ was lower in breeding (17 of 80, 21.2%, 95% CI 12.9 – 31.8) than in non-breeding birds (21 of 42, 50%, 95% CI 34.2 – 65.8; Fig. 1), but this effect was not significant when accounting for sex and age (p = 0.159, Supplementary Table S4). We could not test for an effect of season on prevalence_cloacal_ when including breeding birds, as we did not have any BFDV-negative, young, breeding males. When we confined our analysis to non-breeding birds, we found a significant quadratic effect of date on prevalence_cloacal_ (p = 0.033, Supplementary Table S4), with an increase in prevalence after the breeding season and a subsequent decrease towards the next breeding season. In non-breeding birds, when we tested season instead of date, it was non-significant (p = 0.615, Supplementary Table S4). The effect of date became non-significant, and the model testing season instead of date did not converge, when we included year in the model (Supplementary Table S9).

**Figure 2:**
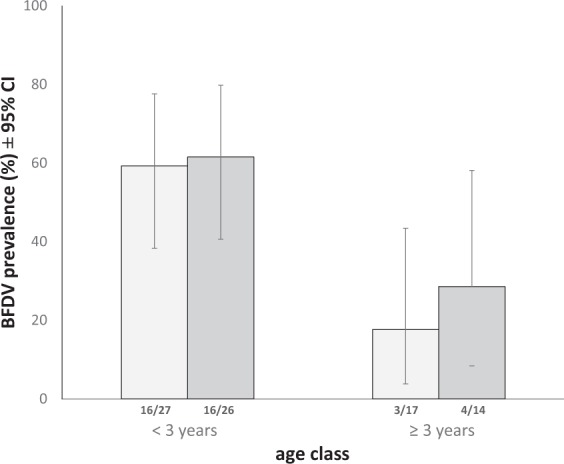
BFDV prevalence (± 95% confidence intervals) shown separately for age classes (<3 years, ≥3 years) and sample types (blood: light grey bars, cloacal swabs: dark grey bars). Shown here: Birds with known age, sampled outside the breeding season, to exclude possible confounding effects of breeding status on BFDV prevalence. Fractions at the base of bars indicate the number of infected birds out of the total number of trapped individuals.

For additional analysis of age effects on sample type, we used only non-breeding birds in order to exclude any possible confounding effect of breeding status (Fig. 2). In non-breeding birds, BFDV prevalence in blood samples was still significantly higher in young birds (<3 years, 16 of 27, 59.3%, 95% CI 38.3 – 77.6) than in older individuals (≥3 years, 3 of 17, 17.7%, 95% CI 3.8 – 43.4; p = 0.01, Supplementary Table S3). We found a trend towards the same difference in cloacal swabs (p = 0.053, Supplementary Table S4).

### Viral load in blood

**Figure 3:**
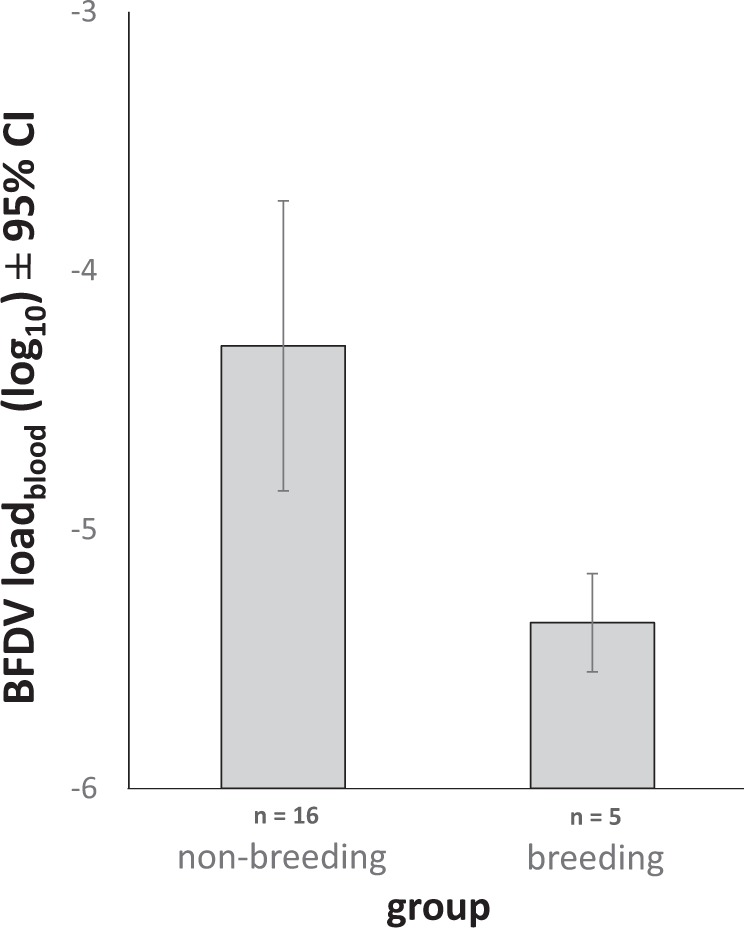
Mean BFDV load, shown separately for non-breeding and breeding birds (‘non-breeding’ denotes birds caught outside the breeding season). Viral load is shown as mean relative gene expression, log_10_ transformed with 95% confidence intervals. Viral load could not be determined from cloacal swabs, due to low DNA yield. Non-breeding birds shown here consist of 14 young (<3 years) and two older (≥3 years) birds, breeding birds were all young birds (<3 years).

Amongst those birds that were BFDV+_blood_, we found that breeding birds had a significantly lower viral load than non-breeding birds (*n* = 21, t = -3.766, df = 16.976, p = 0.002, standard error SE = 0.284; Fig. 3). There was no difference in viral load between sexes in birds that were BFDV+ _blood_ (*n* = 22, t = -0.483, df = 19.643, p = 0.634, SE = 0.46). Our sample size for viral load was insufficient for statistical analysis of seasonal variation and differences between age classes, but we show mean viral load for these categories (Supplementary Fig. S5).

### Seasonal variation in body mass

**Figure 4:**
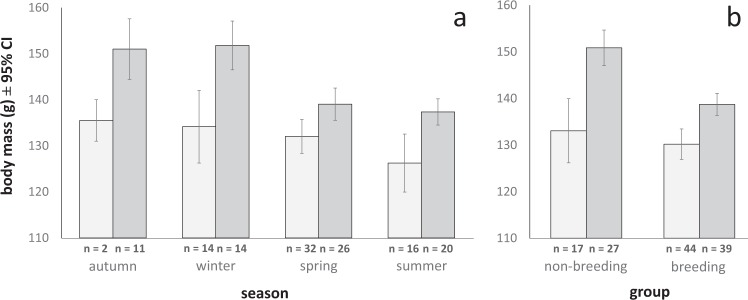
Mean body mass ± 95% confidence intervals shown separately by sex (light grey: females, dark grey: males), season and breeding status (‘non-breeding’ denotes birds caught outside the breeding season).

We found significant seasonal and linear date effects on body mass (Supplementary Table S6), with birds being lightest in spring and summer and heaviest in autumn (Fig. 4). Breeding birds were lighter than non-breeding birds (breeding: *n* = 77, mean m = 134.06 g, 95% CI 131.69 – 136.43, non-breeding: *n* = 42, m = 144.38 g, 95% CI 139.97 – 148.79; p < 0.001, Supplementary Table S6). We found this also when analysing only males (p < 0.001, Supplementary Table S6), but not in females (Fig. 4, p = 0.056, Supplementary Table S6). Using only non-breeding birds, to exclude the possible confounding effect of breeding status, we found no significant effect of BFDV infection (blood: p = 0.271, cloacal swabs: p = 0.055, Supplementary Table S6) or viral load on body mass (p = 0.288, Supplementary Table S6).

## Discussion

Disease outbreaks commonly show seasonal variation^3,5^, yet although BFDV is of major concern for the conservation of psittacines world-wide^12^ and has been studied for decades, no study had tested for seasonal changes in prevalence of BFDV. In wild *P. elegans*, we show that BFDV appears to be present in the host population year-round. Prevalence and viral load varied across the year, and with breeding status and age. In breeding birds, we found a lower BFDV prevalence in blood samples, and lower viral load in blood samples, compared to birds that we caught outside the breeding season. Previous work by our group found a lower than expected BFDV prevalence within breeding *P. elegans* pairs; one possible explanation may be that infected individuals might be less likely to breed^35^. In addition, adult *P. elegans* may be more likely to become infected with BFDV outside of the breeding season because they may be more susceptible after breeding^3^. We provide comparisons of BFDV prevalence during and outside the breeding season, which may suggest, among other explanations, a possible link between infection and the likelihood of breeding.

In our study, we found that BFDV prevalence in both sample types varied with date (i.e. quadratic or cubic date terms, with the cubic date term included where it improved the fit of the model). Prevalence in blood appeared to be highest after the breeding season, in autumn. In cloacal swabs, prevalence seemed to increase after the breeding season and peak in early winter. This later peak in prevalence in cloacal swabs than in blood samples may possibly be due to a delayed onset of viral shedding after a short viraemia^32^. The higher prevalence after the breeding season might be driven by the influx of young birds into the host population following seasonal breeding^32,36,37^. In support of this interpretation, we found that young *P. elegans* had a higher BFDV prevalence in blood than older birds, and a trend towards higher BFDV prevalence in cloacal swabs than the older individuals. Previous studies have also shown that birds under one year of age are particularly likely to get infected with BFDV^33,38,39^. Nestlings may be especially likely to contract BFDV because they may get infected by their parents or by contaminated nesting material^27^; alternatively, they may become infected by conspecifics or interspecific reservoir hosts after the breeding season^37^. In *P. elegans*, nestlings fledge after five weeks and usually accompany the parents for one to two months^40^. They then form post-breeding flocks until the next breeding season^41^, whereas adult *P. elegans* usually stay together in pairs and only rarely form large groups with conspecifics^41,42^. In House Finches (*Carpodacus mexicanus*), flocking behaviour of young birds has been shown to elevate pathogen prevalence, caused by high contact rates with infected individuals^6^ and increased density of susceptible hosts^8^. Young *P. elegans* might be more susceptible to infections, as their immune system is naïve to the virus. Such an effect, with young birds showing lower seroprevalence, higher pathogen prevalence and more intense shedding of pathogens, and being more likely to be infected, has been shown in several avian host-pathogen systems, for example in young *C. mexicanus* that were infected with the bacteria-like *Mycoplasma gallisepticum*^6^, and for infection of wild birds with avian influenza virus^43^. We accounted for host age when testing the effect of date and season, also because our ‘non-breeding birds’ group contained a higher proportion of young birds than the ‘breeding birds’ group, as can be seen in Fig. S1 and Table S1. Our findings reinforce the potential importance of targeted management strategies in threatened species infected with BFDV, in order to protect young birds, because they appear particularly susceptible to BFDV infection. We realise that it would be preferable to enter site and year into the analysis of seasonal variation, to validate the seasonal effect across years and geographical regions. In our study, however, BFDV prevalence for some age or sex groups was so low that the models often did not converge, which is why we could only show some of this information in the Supplementary Material. The bigger sample size we have for one of the years of our study could result in stochastic seasonal variation in that single year leading to an interpretation of overall, consistent seasonal effects. Future studies should include year and site when possible.

In blood samples, we found a BFDV prevalence of 20.0% for males and 24.1% for females. In comparison, previous studies have documented a slightly higher prevalence (males 28.3%, females 30.8%)^35^, although those included other subspecies of *P. elegans*. In *P. e. elegans* alone, 45%^33^ and 34.5%^34^ BFDV prevalence was detected previously in blood, which is again higher than the 21.8% we report in this study. One explanation for the differences between studies may be the effect of breeding status: based on the findings reported here, studies would be expected to find a higher prevalence of BFDV if a higher proportion of birds were sampled outside of the breeding season. In addition to the effects of breeding status, flocking behaviour and age (discussed above), seasonal fluctuations in prevalence of wildlife disease may also be influenced by other variables, including weather factors^3^, which can vary across years, so leading to strong inter-annual fluctuations in prevalence. In contrast to the higher prevalence we detected in females, we found males to be more likely to be infected than females when controlling for season, date, breeding status or age, and when testing non-breeding birds only. We think that our analysis of non-breeding birds may be the most reliable, as subadult male *P. elegans* rarely breed^35^, which can lead to models including breeding birds being quite unbalanced. Including breeding status and age in particular could change the direction of the observed sex effect (females or males more likely to be infected), which should be taken into account when analysing the influence of host sex on BFDV infection. While further research would be desirable, our findings suggest that males may be more likely to be infected with BFDV than females, at least in *P. elegans*.

We found a higher BFDV prevalence in cloacal swabs (33.3%) than in blood samples (21.8%), and this also applied to all subsets of birds we tested (e.g. subsets of breeding, young, old birds). This may indicate that a high proportion of hosts may shed BFDV into the environment, which could have important implications for conservation management. Cloacal swabs are a commonly used method to estimate viral shedding^32,44^, as BFDV and other pathogens are often transmitted via the faecal-oral route^19,45^. Additionally, in birds that were BFDV positive in at least one sample type, we found no association between BFDV presence in blood samples and BFDV presence in cloacal swabs. Some viral infections, such as infection with herpes simplex virus^46^, can become latent, usually after an active phase, and the viruses are then only detectable in certain tissues where they persist until re-activation. BFDV can be undetectable in blood during latent phases of infection, but may still be detectable in the cloaca^32,47^. Positive cloacal swab results in birds with BFDV-negative blood may result from viral persistence within the Bursa of Fabricius, which sits near the cloaca and is one of the main sites of BFDV replication^32,48,49^. On the other hand, birds that are BFDV positive in blood only, but not in cloacal swabs, may be in the early stages of infection, before occurrence of viral shedding^47^. Additionally, a small percentage of the BFDV positives in blood may represent remnant viral DNA from previous infections. It is however thought that the majority of BFDV positives in blood represent active viral infection^35^, and the qPCR detection assay we used is a very widely used method for investigations of BFDV prevalence^33,50^. Cloacal swabs have not been subject to the same level of validation as blood samples for BFDV detection, and cloacal results should therefore be interpreted more cautiously than blood samples. To confirm that the BFDV DNA detected in cloacal swabs is indeed viable virus, and not viral DNA fragments excreted after ingestion, future studies considering shedding of BFDV would benefit from including haemagglutination assays for antigen detection in faecal or feather samples^31^, or sequencing of BFDV-positive swabs, as has been done for blood samples^33^. Our results are, however, in accordance with other studies showing that BFDV^32^ and other circoviruses^48^ can be detected in the cloaca even while not being detectable in blood. As BFDV is thought to be highly stable in the environment^27^, prolonged periods of virus shedding may lead to high levels of environmental contamination. While we cannot fully exclude false negative results due to the much lower DNA yield from swabs than from blood, or false positives due to contamination from feathers surrounding the cloaca, our results suggest that BFDV presence can be missed in subjects if one only tests for active infection in blood.

Lastly, we found no effect of either BFDV infection or viral load on body mass. We did however find seasonal variation in body mass when pooling data from breeding and non-breeding birds. Males in our study had their lowest body mass during the breeding season. Decreased body mass due to chick rearing has been reported in many other avian species^51^, often hypothesised to occur due to increased energy expenditure during the breeding season^3,52^.

## Conclusion

Knowledge of seasonal fluctuations in prevalence and severity of infections, and understanding of their causes, can lead to fundamentally better control of disease risks in wild host populations. Here we discuss that BFDV prevalence in natural *P. elegans* populations can show seasonal variation, with a possible peak prevalence in blood samples occurring after breeding. Our data suggest that this variation may be mainly influenced by changes in host breeding status and the influx of young birds into the population, a finding consistent with studies on other avian pathogens. We detected BFDV in a large proportion of the cloacal swabs we analysed, suggesting that wild hosts might shed the virus over extended periods of time, although swabs should be subject to further validation. Based on our and other data, which show highest prevalence and load in young birds, as well as a higher prevalence_blood_ outside of the breeding season, we conclude that, in *P. elegans* at least, the high-risk time for BFDV infection and transmission might be during and after the breeding season, when young, susceptible host individuals enter the population and subsequently flock together. Accordingly, our findings suggest consideration of targeted BFDV management aimed at the protection of young hosts, at least in threatened or vulnerable species. Future studies should test whether the pattern we find in *P. elegans* is found in other avian hosts of BFDV, and across additional geographical regions and years.

## Methods

### Study species

*P. elegans* is widely distributed throughout south-eastern and eastern Australia^40^. It is classified by the IUCN as of least concern due to its large range and high numbers, but is thought to be declining due to ongoing habitat destruction^53^. *P. elegans* use nest hollows for reproduction and show high nest site fidelity, but low social pair fidelity^54^. Female *P. elegans* can start breeding in their first year, whereas males typically only start breeding when they are at least two years old^55^. The species has been shown to be an excellent model system for BFDV research^33,35,56^.

### Sample collection

We conducted this study under Deakin University animal ethics approval (B31-2015 and B37-2016) and Australian Bird and Bat Banding authority 2319, and it complied with the laws of Victoria (research permit 10007969). We collected samples from *P. e. elegans* in southern Victoria, Australia, from October 2016 until September 2018. We captured breeding birds during two breeding seasons using nest box traps^57^, from October 2016 to January 2017, and from October 2017 to January 2018. We also caught *P. elegans* in baited walk-in cage traps year-round (see Supplementary Table S1 and Supplementary Fig. S1 for information on numbers trapped, by age, sex, season and trap type). We used three field sites: Bellbrae (S38°19′ E144°11′, 110 nest boxes and two walk-in traps), Meredith/She Oaks (S37°51′ E144°06′, 50 nest boxes and two walk-in traps), and Steiglitz (S37°52′ E144°18′, 80 nest boxes and one walk-in trap).

We collected blood samples to study patterns of active infection (prevalence and intensity of infection)^35^, as well as cloacal swabs to estimate viral shedding^32^, and recorded body mass and tarsus length to estimate host condition^51,58,59^. We took ~100 µl of blood from the brachial vein and stored it in ethanol at room temperature. We took cloacal swabs and stored them at 3 °C in the field, then froze them at −80 °C upon return to the laboratory on the same day. To avoid virus transmission between sampled birds, the cotton bags we used to hold birds in were autoclaved after each use, blood sampling equipment was single-use and banding and measuring tools were sprayed with F10 SC Veterinary Disinfectant (Health and Hygiene Pty Ltd, South Africa) after each use.

We assigned birds to one of three age classes based on distinct plumage colouration: subadult (<1 year), young adult (1 – 3 years) and adult (≥3 years)^56^. Nestlings were not included in the study reported here. For analysis, subadults and young adults were combined into the age class ‘young bird’ (<3 years) to ensure an adequate sample size for comparison of breeding and non-breeding birds. To analyse changes in prevalence at a finer scale, we calculated age in months for these ‘young birds’, by calculating the estimated mean fledging time for the 2017 breeding season (*n* = 29 nests, mean 14 Dec, range 12 Nov – 17 Jan). We then correlated capture date with plumage colouration and wing stripe^40^, and assigned age in months accordingly (Supplementary Table S2). For example, we estimated that green subadults with full wing stripe on most or all primaries caught in January were one month old.

### DNA extraction and PCR

To extract BFDV and host DNA from blood and swab samples, we used an ammonium acetate DNA extraction method that is commonly used in BFDV studies and gives high DNA yields^12,33,60^. The extracted DNA was stored in low Tris-EDTA buffer (10 mM Tris.HCL, 0.1 mM EDTA; pH 7.5 – 8.0) at -20 °C^34^. We determined DNA quality and quantity using a DU 640B spectrophotometer (Beckman Coulter, CA, U.S.A.) with a 1: 200 dilution. We sexed birds using a modified PCR protocol by Griffiths, *et al*.^61^. For BFDV detection, we diluted DNA to the same concentration (200 ng/µl), and then used a probe-based quantitative real-time PCR (qPCR) method^34^. We ran the assay using a PikoReal Real-Time PCR System (Thermo Fisher Scientific Inc., MA, U.S.A.). We added positive and no-template controls to each qPCR plate, and all samples were run in duplicate. Duplicate samples with Cq values (cycle at which probe fluorescence crosses the arbitrarily set detection baseline) differing by more than one cycle were run again. Although the qPCR method amplifies a shorter fragment of viral DNA than conventional PCR, it has been shown to deliver comparable results, while being more sensitive than the conventional assay^34^. qPCR is a widely used method for BFDV surveillance^50^. As most positives detected by qPCR represent active infection with viable virus as confirmed by sequencing^33^, but some can be non-active remnant viral DNA, we define prevalence as the percentage of individuals positive for BFDV viral DNA^35^. BFDV-positive (BFDV+) birds are termed either BFDV+_blood_ or BFDV+_cloacal_, depending on which sample type was positive for BFDV presence. Samples that were BFDV-negative are termed BFDV−_blood_ or BFDV−_cloacal_.

For comparative analysis of viral load across individuals, we re-ran BFDV+_blood_ samples we used for comparative viral load analysis on the same qPCR plate, again all at a concentration of 200 ng/µl, to avoid possible slight variation of results across plates. We then used a comparative method to calculate viral load using the Cq values of each BFDV-positive sample^33,62^: Viral load = 2^(−ΔCq)^. The resulting data were then log_10_-transformed to achieve normality^34^. Diluting cloacal swab DNA to the same concentration was not possible due to much lower DNA yield. We therefore only report prevalence for cloacal swabs, and not viral load. We re-tested a subset of walk-in-trapped *P. elegans* blood samples (subset with the highest BFDV prevalence) which were BFDV−_blood_, to estimate the likelihood of false negatives. None of the samples which were initially BFDV−_blood_ came up BFDV+_blood_ in the repeat run.

### Statistical analyses

We carried out statistical analyses using SPSS 25.01 (IBM, Armonk NY, U.S.A.). To ensure independence of cases, we used only data from the first capture if individuals were recaptured. We pooled the data from our two years of study and from the three field sites, because we found overlapping confidence intervals between years and sites during initial data analysis (Supplementary Fig. S6). Additionally, Eastwood, *et al*.^33^ found that in *P. elegans*, geographic location, host density and parrot community diversity and composition do not explain differences in BFDV prevalence or load. A very low BFDV prevalence in some sex/age groups did not permit us to include year and trapping site as random intercepts, as well as age and sex in the same model, as the models did not converge. When we ran site and year separately as main effects in the full models, they were not significant, while we still found effects of age, date and sex (Supplementary Tables S7 – S10). We combined two of our three field sites, namely Meredith/She Oaks and Steiglitz, for statistical analysis. The two sites are located within 10 km of each other. Data from previous studies show that most recaptured *P. elegans* were trapped or resighted within approximately 10 km of their banding site^54,63^. We therefore defined Meredith/She Oaks and Steiglitz as one *P. elegans* population. We are aware that due to different and sometimes low sample sizes of infected birds trapped in some years and sites, and the resulting grouping of years and sites for most of our analyses, some of the observed seasonal patterns may in fact be stochastic effects. We address this in the discussion.

Initial calculations of prevalence included birds trapped during the breeding season (September – January, caught as parental birds in nest box, hereafter referred to as ‘breeding’) and birds trapped outside the breeding season (February – August, caught in walk-in traps, hereafter referred to as ‘non-breeding’), to represent an average prevalence across the population^35^. We report prevalence by sample type tested, as prevalence_blood_ (refers to population prevalence in blood samples) and prevalence_cloacal_ (refers to population prevalence in cloacal swabs).

We analysed BFDV prevalence and host body mass using generalized linear models (GLM), which are commonly used as a flexible method for analysis of seasonal variation^64^. For binary data, we report Binomial (Clopper-Pearson) ‘exact’ CI as a widely used, conservative method recommended for small sample sizes^65^. We used a binomial distribution with a logit link to analyse effects on prevalence and a Gaussian distribution with identity link for the analysis of body condition and body mass. Where applicable, we checked the residuals to confirm that they conformed to the model assumptions. We report model outputs in Supplementary Tables S3–S11. In all figures and the main body of the text, we show raw data, i.e. means with 95% confidence intervals (CI). To report model fit values for binary data (e.g. infection status) we used the Nagelkerke R^2^. For continuous data (e.g. body mass), we show the overall R^2^ created by univariate analysis of variance.

We analysed temporal changes in BFDV prevalence using two predictors: season (spring, summer, autumn, winter) and Julian date (‘date’; with the 1^st^ February set as day 1). We analysed date as a continuous variable in addition to season, because grouping samples by season can result in samples that were in fact collected close together being assigned to different seasons, potentially leading to unreliable results (for example, a bird trapped on the 31^st^ May would be in the autumn group, but a bird trapped one day later would be in the winter group). Both season (as a categorical predictor) and date (as a continuous predictor) are common methods for investigating seasonal effects^64,66^. Seasons were defined as follows: Spring: 1^st^ September to 30^th^ November; summer: 1^st^ December to 28^th^ February; autumn: 1^st^ March to 31^st^ May; winter: 1^st^ June to 31^st^ August. Season was included as an ordinal variable (season 1 = spring, 2 = summer, 3 = autumn, 4 = winter). During exploratory data analysis we ran the same models using season as either ordinal or nominal variable, which led to identical results. Julian date was set to day one on the 1^st^ February, to represent a biologically significant date^67^, i.e. the end of the breeding season of *P. elegans* in our study area^35^. During exploratory data analysis, we also tested 1^st^ January (start of the year) and 1^st^ September (beginning of the breeding season) as Julian day 1. Between the three examples of Julian day 1, the resulting R^2^, AICc and p-values were very similar (Supplementary Table S11); we therefore only report results based on 1^st^ February as day 1. We centred Julian date to avoid collinearity, and tested linear, quadratic and cubic date predictors. For blood samples, models with quadratic date terms showed better fit (based on AICc and Nagelkerke R^2^) than models with cubic as well as quadratic date terms (data not shown). For body condition and body mass, models had almost identical fit with and without the cubic date terms. We therefore chose to use the most parsimonious models and for blood samples, body condition and body mass, we report model outputs without the cubic date term. For cloacal swabs and when testing our complete data set *of P. elegans*, models including cubic as well as quadratic date terms showed better fit than models that included only quadratic terms. We thus report results based on these models (Supplementary Table S4). Additional predictors we used were host traits that have previously been shown to influence BFDV infection (age class and sex)^56^. After performing GLMs on the full data set, we also tested seasonal effects separately in subsets of breeding and non-breeding birds, as date and breeding status were correlated. Nine birds which were caught in walk-in traps during the breeding season were excluded from analyses comparing breeding and non-breeding birds, as we could not determine whether or not they were breeding at the time of capture. Sample size thus varies between models, and also because of missing information for some predictors for some individual birds.

We repeated models analysing body mass by including tarsus length as a predictor, to estimate body condition by accounting for individual size differences. Body condition results are only shown in Supplementary Table S5, as they are very similar to results with body mass which did not include tarsus length (Supplementary Table S6). To compare viral load between groups (males vs females, breeding vs non-breeding) we conducted t-tests^35^.

## Supplementary information

Supplementary information.
