# Supplementary material for: Seasonal fluctuation of beak and feather disease virus (BFDV) infection in wild Crimson Rosellas (Platycercus elegans)
Source: Sci Rep. 2020 May 12;10:7894. doi: 10.1038/s41598-020-64631-y (PMC7217931; doi:10.1038/s41598-020-64631-y)
Supplement: Supplementary file 1 — Supplementary information. [file 41598_2020_64631_MOESM1_ESM.docx]

**Supplementary Material**

**Seasonal fluctuation of beak and feather disease virus (BFDV) infection in wild Crimson Rosellas (*Platycercus elegans*)**

Johanne M. Martens^1,^*, Helena S. Stokes^1^, Mathew L. Berg^1^, Ken Walder^2^, Andy T. D. Bennett^1^

^1^Centre for Integrative Ecology, Deakin University, 75 Pigdons Road, Waurn Ponds VIC 3216, Australia

^2^Centre for Molecular and Medical Research, School of Medicine, Deakin University, 75 Pigdons Road, Waurn Ponds VIC 3216, Australia

*corresponding author, [j.martens@deakin.edu.au](mailto:j.martens@deakin.edu.au)

**Supplementary figures**

**Supplementary Figure S1:** Age and sex ratios of birds trapped, shown by season and breeding status. Light grey bars represent males; dark grey bars represent females. Only birds with known sex and age are shown. Nine birds which were caught in walk-in traps during the breeding season are not shown, as we cannot determine whether or not they were breeding and therefore excluded them from analyses comparing breeding and non-breeding birds. Numbers at the base and top of bars indicate number of birds of shown sex caught, out of total number caught during the season shown.

**Supplementary Figure S2:** Mean BFDV prevalence in a) blood and b) cloacal swabs ± 95% confidence intervals for different subsets of females (dark grey) and males (light grey). Category ‘all’ includes breeding and non-breeding birds, as well as birds with unknown breeding status, of all ages. Numbers at the base of bars indicate number of BFDV+ birds out of total number of birds tested. Where prevalence is 0%, one-sided 97.5% confidence intervals are shown.

**Supplementary Figure S3:** Mean BFDV prevalence (%) with 95% confidence intervals in blood samples in young (< 3 years) females only. BFDV prevalence is shown for each season for walk-in trapped birds (labelled with the seasons) and separately for breeding birds (‘spring/summer breeding’). Numbers at the base of bars indicate number of infected out of number of trapped individuals. Where prevalence is 0%, one-sided 97.5% confidence intervals are shown.

**Supplementary Figure S4:** Mean BFDV prevalence in young birds (< 3 years) by age category, with 95% confidence intervals. a) BFDV prevalence in blood samples and b) in cloacal swabs. Numbers at the base of bars indicate the number of infected birds out of total number of birds tested. Age categories represent age in months pooled into 6 categories: 1 (1 – 6 months), 2 (7 – 12 months), 3 (13 – 18 months), 4 (19 – 24 months), 5 (25 – 36 months). Where prevalence is 0%, one-sided 97.5% confidence intervals are shown.

**Supplementary Figure S5:** Mean viral load, shown as log_10_-transformed relative gene expression ± 95 % confidence intervals by a) season and b) age in months, for birds that were up to 24 months old. Seasons ‘summer’ to ‘winter’ show mean viral load of birds caught in walk-in traps, ‘spring-summer breeding’ shows results for breeding birds caught in nest boxes. Age in months is pooled into categories: category 1 includes birds that are one to six months old, 2 contains birds of seven to 12 months, 3 includes 13 – 18 months old birds, and 4 includes birds of 19 – 24 months.

**Supplementary Figure S6:** Mean BFDV prevalence by year and field site, ± 95% confidence intervals. By year: a) in blood samples, b) in cloacal swabs. Dark grey bars represent year 1, light grey bars represent year 2. By field site: c) in blood samples, d) in cloacal swabs. Dark grey bars represent field site Bellbrae, intermediate grey bars represent Meredith/She Oaks, and light grey bars represent Steiglitz. In Steiglitz, we did not trap birds outside the breeding season.

**Supplementary tables**

**Supplementary Table S1:** Summary of sample sizes of P. elegans caught for each age class, sex and trap type.

| **subset** | **young birds (< 3 years)** | **adults (≥ 3 years)** | **unknown age** |
| --- | --- | --- | --- |
| All (*n* = 142) | 48 (33 ♀, 15 ♂) | 84 (29 ♀, 55 ♂) | 10 (5 ♀, 5 ♂) |
| Caught in walk-in traps (*n* = 55) | 27 (14 ♀, 13 ♂) | 26 (8 ♀, 18 ♂) | 2 (2 ♂) |
| Caught in nest boxes (*n* = 87) | 21 (19 ♀, 2 ♂) | 58 (21 ♀, 37 ♂) | 8 (5 ♀, 3 ♂) |

**Supplementary Table S2:** Changes in plumage colouration in young P. elegans^40^. Fledglings leave the nest with broad stripes (‘full stripes’) on the underwing feathers (primaries), which during moult get replaced first by narrower stripes (‘half stripes’), then by dark feathers without stripes. Birds older than 23 months of age could not be aged by month, but only by year.

| **age** | **time of moult** | **moult details** | **result of moult** | **age assigned by plumage colouration** |
| --- | --- | --- | --- | --- |
| Subadult (< 1 year) | October to March  of 1^st^ year | Prolonged. Partial, most plumage, not remiges and retrices. | Parts of the contour feather red.  Most primaries still with ‘full stripes’. | Green body & full stripe: 1 – 8 months.  Red-green mottled & ‘full stripe’ on primaries: 8 – 16 months |
| 2^nd^ year | September to November  of 2^nd^ year | Largely complete, includes primaries. | Red contour feathers  Few to no primaries with ‘full’ stripe. These replaced by primaries with ‘half stripe’. | Mostly red, some half stripes, few or no ‘full stripes’: 21 – 23 months |
| 3^rd^ year onwards | Post-breeding (November onwards) | Complete | Red contour feathers, no wing stripes | Red, no wing stripe: month 24 onwards. |

**Supplementary Table S3:** The effects on BFDV prevalence in blood samples of P. elegans. We tested host sex, host age, season, date or breeding status as predictors which may influence prevalence. Subset ‘all’ contains all birds tested regardless of age, sex and breeding status; subset ‘non-breeding’ contains birds that were caught outside the breeding season in walk-in traps. Predictor ‘breeding status’ refers to whether birds were considered ‘non-breeding’ or ‘breeding’ (i.e. were caught during the breeding season in nest boxes). Significant results are shown in bold print. Sample sizes vary due to use of different subsets of birds, and because some sample types could not be obtained from some individuals.

| **subset** | ***n* birds** | **predictor** |  | **OR** | **95% CI for OR** | **Wald χ^2^** | **df** | **p** | **R^2^** |
| --- | --- | --- | --- | --- | --- | --- | --- | --- | --- |
| all | 132 | season (winter) | spring | 0.325 | 0.101 – 1.038 | 6.762 | 3 | 0.08 | 0.429 |
|  |  |  | summer | 0.164 | 0.037 – 0.719 |  |  |  |  |
|  |  |  | autumn | 0.289 | 0.042 – 1.988 |  |  |  |  |
|  |  | sex (female) | male | 6.124 | 1.535 – 24.439 | 6.587 | 1 | **0.01** |  |
|  |  | age (< 3 yrs) | ≥ 3 yrs | 0.028 | 0.006 – 0.130 | 20.663 | 1 | **< 0.001** |  |
|  | 132 | date |  | 1.011 | 1.003 – 1.019 | 7.871 | 1 | **0.005** | 0.441 |
|  |  | date^2^ |  |  |  | 3.993 | 1 | **0.046** |  |
|  |  | sex (female) | male | 4.493 | 1.098 – 18.380 | 4.37 | 1 | **0.037** |  |
|  |  | age (< 3 yrs) | ≥ 3 yrs | 0.032 | 0.006 – 0.154 | 18.305 | 1 | **< 0.001** |  |
|  | 123 | (breeding) | non-breeding | 3.946 | 1.312 – 11.870 | 5.968 | 1 | **0.015** | 0.463 |
|  |  | sex (female) | male | 4.467 | 1.009 – 19.773 | 3.889 | 1 | **0.049** |  |
|  |  | age (< 3 yrs) | ≥ 3 yrs | 0.027 | 0.005 – 0.153 | 16.765 | 1 | **< 0.001** |  |
| non-breeding | 44 | season (winter) | summer | 0.556 | 0.027 – 11.364 | 2.101 | 2 | 0.35 | 0.388 |
|  |  |  | autumn | 0.237 | 0.034 – 1.667 |  |  |  |  |
|  |  | sex (female) | male | 9.612 | 1.272 – 72.665 | 4.808 | 1 | **0.028** |  |
|  |  | age (< 3 yrs) | ≥ 3 yrs | 0.046 | 0.006 – 0.381 | 8.142 | 1 | **0.004** |  |
|  | 44 | date |  | 0.967 | 0.901 – 1.038 | 0.843 | 1 | 0.359 | 0.371 |
|  |  | date^2^ |  |  |  | 0.515 | 1 | 0.473 |  |
|  |  | sex (female) | male | 8.769 | 1.238 – 62.114 | 4.725 | 1 | **0.03** |  |
|  |  | age (< 3 yrs) | ≥ 3 yrs | 0.047 | 0.006 – 0.382 | 8.211 | 1 | **0.004** |  |
|  | 44 | sex (female) | male | 6.180 | 1.034 – 36.955 | 3.985 | 1 | **0.046** | 0.335 |
|  |  | age (< 3 yrs) | ≥ 3 yrs | 0.051 | 0.007 – 0.358 | 8.987 | 1 | **0.003** |  |
|  | 44 | age (< 3 yrs) | ≥ 3 yrs | 0.147 | 0.034 – 0.637 | 6.571 | 1 | **0.01** | 0.219 |

Models with quadratic date terms only are shown, as they showed better fit (based on AICc and Nagelkerke R^2^) than models with quadratic cubic as well as quadratic date terms. Effect sizes are given as odds ratio (OR), where an OR > 1 indicates that the given category is more likely to be infected than the reference category. Reference categories are shown in parentheses.

**Supplementary Table S4:** The effects on BFDV prevalence in cloacal swabs of P. elegans. We tested host sex, host age, season, date or breeding status as predictors which may influence prevalence. Subset ‘all’ contains all birds tested regardless of age, sex and breeding status; subset ‘non-breeding’ contains birds that were caught outside the breeding season in walk-in traps. Predictor ‘breeding status’ refers to whether birds were considered ‘non-breeding’ or ‘breeding’ (i.e. were caught during the breeding season in nest boxes). Significant results are shown in bold print. Sample sizes vary due to use of different subsets of birds, and because some sample types could not be obtained from some individuals.

| **subset** | ***n* birds** | **predictor** |  | **OR** | **95% CI for OR** | **Wald χ^2^** | **df** | **p** | **R^2^** |
| --- | --- | --- | --- | --- | --- | --- | --- | --- | --- |
| all | 120 | blood (not infected) | infected | 2.728 | 0.850 – 8.756 | 2.846 | 1 | 0.092 | 0.410 |
|  |  | date |  | 1.014 | 1.004 – 1.025 | 7.157 | 1 | **0.007** |  |
|  |  | date^2^ |  |  |  | 3.422 | 1 | 0.064 |  |
|  |  | date^3^ |  |  |  | 6.654 | 1 | **0.01** |  |
|  |  | sex (female) | male | 1.339 | 0.321 – 5.590 | 0.16 | 1 | 0.689 |  |
|  |  | age (< 3 yrs) | ≥ 3 yrs | 0.101 | 0.023 – 0.446 | 9.141 | 1 | **0.002** |  |
|  | 120 | date |  | 1.015 | 1.004 – 1.025 | 7.817 | 1 | **0.005** | 0.386 |
|  |  | date^2^ |  |  |  | 2.325 | 1 | 0.127 |  |
|  |  | date^3^ |  |  |  | 5.662 | 1 | **0.017** |  |
|  |  | sex (female) | male | 1.817 | 0.448 – 7.379 | 0.698 | 1 | 0.403 |  |
|  |  | age (< 3 yrs) | ≥ 3 yrs | 0.061 | 0.015 – 0.253 | 14.906 | 1 | **< 0.001** |  |
|  | 114 | (breeding) | non-breeding | 2.000 | 0.762 – 5.248 | 1.981 | 1 | 0.159 | 0.306 |
|  |  | sex (female) | male | 1.562 | 0.467 – 5.229 | 0.523 | 1 | 0.469 |  |
|  |  | age (< 3 yrs) | ≥ 3 yrs | 0.104 | 0.030 – 0.363 | 12.576 | 1 | **< 0.001** |  |
| non-breeding | 40 | date |  | 1.108 | 0.921 – 1.333 | 1.186 | 1 | 0.276 | 0.311 |
|  |  | date^2^ |  |  |  | 0.551 | 1 | 0.458 |  |
|  |  | date^3^ |  |  |  | 0.184 | 1 | 0.668 |  |
|  |  | sex (female) | male | 0.777 | 0.125 – 4.828 | 0.073 | 1 | 0.786 |  |
|  |  | age (< 3 yrs) | ≥ 3 yrs | 0.143 | 0.021 – 0.980 | 3.923 | 1 | **0.048** |  |
|  | 40 | season (winter) | summer | 0.268 | 0.020 – 3.676 | 0.973 | 2 | 0.615 | 0.163 |
|  |  |  | autumn | 0.874 | 0.181 – 4.219 |  |  |  |  |
|  |  | sex (female) | male | 0.880 | 0.180 – 4.290 | 0.025 | 1 | 0.874 |  |
|  |  | age (< 3 yrs) | ≥ 3 yrs | 0.237 | 0.045 – 1.257 | 2.862 | 1 | 0.091 |  |
|  | 40 | sex (female) | male | 0.753 | 0.169 – 3.363 | 0.138 | 1 | 0.710 | 0.133 |
|  |  | age (< 3 yrs) | ≥ 3 yrs | 0.288 | 0.059 – 1.396 | 2.391 | 1 | 0.122 |  |
|  | 40 | age (< 3 yrs) | ≥ 3 yrs | 0.250 | 0.061 – 1.017 | 3.75 | 1 | 0.053 | 0.129 |

For cloacal swabs, models including cubic as well as quadratic date terms are shown, as they showed better fit (based on AICc and Nagelkerke R^2^) than models that included only quadratic date terms. Effect sizes are given as odds ratio (OR), where an OR > 1 indicates that the given category is more likely to be infected than the reference category. Reference categories are shown in parentheses.

**Supplementary Table S5:** The effects on body condition of P. elegans. We tested infection status (BFDV presence or absence in blood samples and/or cloacal swabs), host sex, host age, season, date or breeding status as predictors which may influence prevalence. Subset ‘all’ contains all birds tested regardless of age, sex and breeding status; subset ‘non-breeding’ contains birds that were caught outside the breeding season in walk-in traps. Predictor ‘breeding status’ refers to whether birds were considered ‘non-breeding’ or ‘breeding’ (i.e. were caught during the breeding season in nest boxes). Significant results are shown in bold print. BFDV infection status was only tested as a predictor in non-breeding birds, to exclude confounding effects of breeding status. Sample sizes vary due to use of different subsets of birds, and because some sample types could not be obtained from some individuals.

| **subset** | ***n* birds** | **predictor** |  | **B-value** | **95% CI for B** | **Wald χ^2^** | **df** | **p** | **R^2^** |
| --- | --- | --- | --- | --- | --- | --- | --- | --- | --- |
| all | 112 | season (winter) | spring | -5.678 | -10.232, -1.125 | 33.392 | 3 | **< 0.001** | 0.504 |
|  |  |  | summer | -11.543 | -16.365, -6.720 |  |  |  |  |
|  |  |  | autumn | 4.376 | -2.392, 11.144 |  |  |  |  |
|  |  | sex (male) | female | -7.679 | -12.022, -3.336 | 12.012 | 1 | **0.001** |  |
|  |  | age (≥ 3 yrs) | < 3 yrs | -4.527 | -8.493, -0.561 | 5.005 | 1 | **0.025** |  |
|  |  | tarsus |  | 3.406 | 1.075, 5.737 | 8.201 | 1 | **0.004** |  |
|  | 112 | date |  | -0.068 | -0.094, -0.042 | 26.254 | 1 | **< 0.001** | 0.480 |
|  |  | date^2^ |  | 0.000 | -0.001, -3.855E-5 | 5.063 | 1 | **0.024** |  |
|  |  | sex (male) | female | -6.059 | -10.621, -1.497 | 6.775 | 1 | **0.009** |  |
|  |  | age (≥ 3 yrs) | < 3 yrs | -5.542 | -9.760, -1.323 | 6.629 | 1 | **0.01** |  |
|  |  | tarsus |  | 4.183 | 1.842, 6.524 | 12.262 | 1 | **< 0.001** |  |
|  | 105 | (non-breeding) | breeding | -9.530 | -13.464, -5.595 | 22.532 | 1 | **< 0.001** | 0.494 |
|  |  | sex (male) | female | -7.018 | -11.738, -2.298 | 8.494 | 1 | **0.004** |  |
|  |  | age (≥ 3 yrs) | < 3 yrs | -6.581 | -10.876, -2.286 | 9.02 | 1 | **0.003** |  |
|  |  | tarsus |  | 3.496 | 0.990, 6.003 | 7.477 | 1 | **0.006** |  |
| non-breeding | 32 | blood (infected) | not infected | 4.417 | -0.830, 9.664 | 2.723 | 1 | 0.099 | 0.748 |
|  |  | cloacal (infected) | not infected | 2.890 | -2.199, 7.979 | 1.239 | 1 | 0.266 |  |
|  |  | sex (male) | female | -19.114 | -25.516, -12.712 | 34.243 | 1 | **< 0.001** |  |
|  |  | age (≥ 3 yrs) | < 3 yrs | 0.099 | -6.389, 6.587 | 0.001 | 1 | 0.976 |  |
|  |  | tarsus |  | 4.834 | 1.918, 7.750 | 10.556 | 1 | **0.001** |  |
|  | 13 | viral load |  | -1.044 | -3.381,1.294 | 0.766 | 1 | 0.381 | 0.902 |
|  |  | sex (male) | female | -26.562 | -32.787, -20.337 | 69.949 | 1 | **< 0.001** |  |
|  |  | age (≥ 3 yrs) | < 3 yrs | 7.472 | -0.211, 15.155 | 3.633 | 1 | 0.057 |  |
|  |  | tarsus |  | 4.375 | 1.115, 7.635 | 6.917 | 1 | **0.009** |  |

Models had almost identical fit (based on R^2^ and AICc values) with and without a cubic date term in addition to the quadratic date term. We therefore chose to report only the most parsimonious models, without the cubic date term. Effect sizes are given as B-values. Reference categories are shown in parentheses.

**Supplementary Table S6:** The effects on body mass of P. elegans. We tested infection status (BFDV presence or absence in blood samples and/or cloacal swabs), host sex, host age, season, date or breeding status as predictors which may influence prevalence. Subset ‘all’ contains all birds tested regardless of age, sex and breeding status; subset ‘non-breeding’ contains birds that were caught outside the breeding season in walk-in traps. Predictor ‘breeding status’ refers to whether birds were considered ‘non-breeding’ or ‘breeding’ (i.e. were caught during the breeding season in nest boxes). Significant results are shown in bold print. BFDV infection status was only tested as a predictor in non-breeding birds, to exclude confounding effects of breeding status. Sample sizes vary due to use of different subsets of birds, and because some sample types could not be obtained from some individuals.

| **subset** | ***n* birds** | **Predictor** |  | **B-value** | **95% CI for B** | **Wald χ^2^** | **df** | **p** | **R^2^** |
| --- | --- | --- | --- | --- | --- | --- | --- | --- | --- |
| all | 127 | season (winter) | spring | -8.676 | -13.148, -4.205 | 41.183 | 3 | **< 0.001** | 0.434 |
|  |  |  | summer | -12.761 | -17.616, -7.907 |  |  |  |  |
|  |  |  | autumn | 3.710 | -3.064, 10.485 |  |  |  |  |
|  |  | sex (male) | female | -8.320 | -12.387, -4.254 | 16.084 | 1 | **< 0.001** |  |
|  |  | age (≥ 3 yrs) | < 3 yrs | -6.010 | -10.081, -1.940 | 8.375 | 1 | **0.004** |  |
|  | 127 | date |  | -0.074 | -0.100, -0.047 | 30.32 | 1 | **< 0.001** | 0.396 |
|  |  | date^2^ |  | 0.000 | -0.001, -5.506E-5 | 5.512 | 1 | **0.019** |  |
|  |  | sex (male) | female | -7.744 | -12.021, -3.467 | 12.594 | 1 | **< 0.001** |  |
|  |  | age (≥ 3 yrs) | < 3 yrs | -6.910 | -11.259, -2.562 | 9.7 | 1 | **0.002** |  |
|  | 119 | (non-breeding) | breeding | -11.087 | -14.950, -7.225 | 31.649 | 1 | **< 0.001** | 0.436 |
|  |  | sex (male) | female | -8.063 | -12.373, -3.754 | 13.448 | 1 | **< 0.001** |  |
|  |  | age (≥ 3 yrs) | < 3 yrs | -7.760 | -12.156, -3.364 | 11.97 | 1 | **0.001** |  |
| non-breeding | 38 | blood (infected) | not infected | 3.866 | -3.024, 10.756 | 1.209 | 1 | 0.271 | 0.556 |
|  |  | cloacal (infected) | not infected | 6.357 | -0.136, 12.851 | 3.682 | 1 | 0.055 |  |
|  |  | sex (male) | female | -14.658 | -22.203, -7.114 | 14.5 | 1 | **< 0.001** |  |
|  |  | age (≥ 3 yrs) | < 3 yrs | -5.531 | -13.969, 2.906 | 1.651 | 1 | 0.199 |  |
|  | 15 | viral load |  | -0.766 | -4.251, 2.720 | 0.185 | 1 | 0.667 | 0.749 |
|  |  | sex (male) | female | -26.232 | -34.009, -18.456 | 43.715 | 1 | **< 0.001** |  |
|  |  | age (≥ 3 yrs) | < 3 yrs | 7.896 | -3.932, 19.725 | 1.712 | 1 | 0.191 |  |
| females (all ages) | 57 | (non-breeding) | breeding | -5.912 | -11.986, 0.162 | 3.64 | 1 | 0.056 | 0.218 |
|  |  | age (≥ 3 yrs) | < 3 yrs | -12.314 | -18.624, -6.003 | 14.628 | 1 | **< 0.001** |  |
|  | 57 | season (winter) | spring | -4.964 | -11.529, 1.602 | 9.399 | 3 | **0.024** | 0.286 |
|  |  |  | summer | -10.691 | -18.056, -3.326 |  |  |  |  |
|  |  |  | autumn | 3.169 | -11.656, 17.993 |  |  |  |  |
|  |  | age (≥ 3 yrs) | < 3 yrs | -12.680 | -18.697, -6.662 | 17.058 | 1 | **< 0.001** |  |
| males  (all ages) | 62 | (non-breeding) | breeding | -14.321 | -18.649, -9.992 | 42.05 | 1 | **< 0.001** | 0.424 |
|  |  | age (≥ 3 yrs) | < 3 yrs | -2.961 | -8.335, 2.413 | 1.166 | 1 | 0.28 |  |
|  | 62 | season (winter) | spring | -10.883 | -16.388, -5.378 | 48.139 | 3 | **< 0.001** | 0.456 |
|  |  |  | summer | -14.352 | -19.654, -9.049 |  |  |  |  |
|  |  |  | autumn | 2.721 | -3.634, 9.076 |  |  |  |  |
|  |  | age (≥ 3 yrs) | < 3 yrs | 0.302 | -4.737, 5.340 | 0.014 | 1 | 0.907 |  |

Models had almost identical fit (based on R^2^ and AICc values) with and without a cubic date term in addition to the quadratic date term. We therefore chose to report only the most parsimonious models, without the cubic date term. Effect sizes are given as B-values. Reference categories are shown in parentheses.

**Supplementary Table S7:** The effects on BFDV prevalence in blood samples of P. elegans (as reported in Supplementary Table S3), whilst controlling for calendar year of sampling. Subset ‘all’ contains all birds tested regardless of age, sex and breeding status; subset ‘non-breeding’ contains birds that were caught outside the breeding season in walk-in traps. Predictor ‘breeding status’ refers to whether birds were considered ‘non-breeding’ or ‘breeding’ (i.e. were caught during the breeding season in nest boxes). Significant results are shown in bold print. Sample sizes vary due to use of different subsets of birds, and because some sample types could not be obtained from some individuals.

| **subset** | ***n* birds** | **predictor** |  | **OR** | **95% CI for OR** | **Wald χ^2^** | **df** | **p** | **R^2^** |
| --- | --- | --- | --- | --- | --- | --- | --- | --- | --- |
| all | 132 | season (winter) | spring | 0.326 | 0.092 – 1.148 | 5.119 | 3 | 0.163 | 0.430 |
|  |  |  | summer | 0.160 | 0.092 – 0.903 |  |  |  |  |
|  |  |  | autumn | 0.272 | 0.038 – 1.938 |  |  |  |  |
|  |  | sex (female) | male | 5.418 | 1.205 – 24.366 | 4.852 | 1 | **0.028** |  |
|  |  | age (< 3 yrs) | ≥ 3 yrs | 0.032 | 0.006 – 0.174 | 15.788 | 1 | **< 0.001** |  |
|  |  | year (2018) | 2016 | 0.652 | 0.066 – 6.452 | 0.137 | 2 | 0.934 |  |
|  |  |  | 2017 | 0.712 | 0.091 – 5.556 |  |  |  |  |
|  | 132 | date |  | 1.012 | 1.002 – 1.022 | 6.073 | 1 | **0.014** | 0.443 |
|  |  | date^2^ |  |  |  | 2.304 | 1 | 0.129 |  |
|  |  | sex (female) | male | 4.907 | 1.079 – 22.308 | 4.238 | 1 | **0.04** |  |
|  |  | age (< 3 yrs) | ≥ 3 yrs | 0.028 | 0.005 – 0.160 | 16.125 | 1 | **< 0.001** |  |
|  |  | year (2018) | 2016 | 1.739 | 0.086 – 35.714 | 0.175 | 2 | 0.916 |  |
|  |  |  | 2017 | 1.300 | 0.094 – 17.857 |  |  |  |  |
|  | 123 | (breeding) | non-breeding | 4.985 | 1.221 – 20.353 | 5.008 | 1 | **0.025** | 0.467 |
|  |  | sex (female) | male | 5.070 | 1.066 – 24.107 | 4.164 | 1 | **0.041** |  |
|  |  | age (< 3 yrs) | ≥ 3 yrs | 0.022 | 0.004 – 0.142 | 16.310 | 1 | **< 0.001** |  |
|  |  | year (2018) | 2016 | 2.625 | 0.162 – 41.667 | 0.462 | 2 | 0.794 |  |
|  |  |  | 2017 | 1.898 | 0.214 – 16.950 |  |  |  |  |
| non-breeding | 44 | season (winter) | summer | 0.328 | 0.003 – 40.000 | 2.039 | 2 | 0.361 | 0.390 |
|  |  |  | autumn | 0.207 | 0.023 – 1.848 |  |  |  |  |
|  |  | sex (female) | male | 9.601 | 1.280 – 71.992 | 4.842 | 1 | **0.028** |  |
|  |  | age (< 3 yrs) | ≥ 3 yrs | 0.048 | 0.006 – 0.411 | 7.694 | 1 | **0.006** |  |
|  |  | year (2018) | 2017 | 0.580 | 0.013 – 26.316 | 0.079 | 1 | 0.779 |  |
|  | 44 | date |  | 0.980 | 0.886 – 1.084 | 0.155 | 1 | 0.694 | 0.373 |
|  |  | date^2^ |  |  |  | 0.004 | 1 | 0.950 |  |
|  |  | sex (female) | male | 9.212 | 1.248 – 68.013 | 4.739 | 1 | **0.029** |  |
|  |  | age (< 3 yrs) | ≥ 3 yrs | 0.047 | 0.006 – 0.387 | 8.065 | 1 | **0.005** |  |
|  |  | year (2018) | 2017 | 0.241 | 9.278E-5 – 500.000 | 0.126 | 1 | 0.723 |  |
|  | 44 | age (< 3 yrs) | ≥ 3 yrs | 0.148 | 0.033 – 0.671 | 6.142 | 1 | **0.013** | 0.219 |
|  |  | year (2018) | 2017 | 0.963 | 0.133 – 6.993 | 0.001 | 1 | 0.970 |  |

Models with quadratic date terms only are shown, as they showed better fit (based on AICc and Nagelkerke R^2^) than models with quadratic cubic as well as quadratic date terms. Effect sizes are given as odds ratio (OR), where an OR > 1 indicates that the given category is more likely to be infected than the reference category. Reference categories are shown in parentheses.

**Supplementary Table S8:** The effects on BFDV prevalence in blood samples of P. elegans (as reported in Supplementary Table S3), whilst controlling for site (field site where samples were collected). Subset ‘all’ contains all birds tested regardless of age, sex and breeding status; subset ‘non-breeding’ contains birds that were caught outside the breeding season in walk-in traps. Predictor ‘breeding status’ refers to whether birds were considered ‘non-breeding’ or ‘breeding’ (i.e. were caught during the breeding season in nest boxes). Significant results are shown in bold print. Sample sizes vary due to use of different subsets of birds, and because some sample types could not be obtained from some individuals.

| **subset** | ***n* birds** | **predictor** |  | **OR** | **95% CI for OR** | **Wald χ^2^** | **df** | **p** | **R^2^** |
| --- | --- | --- | --- | --- | --- | --- | --- | --- | --- |
| all | 132 | season (winter) | spring | 0.323 | 0.101 – 1.035 | 6.557 | 3 | 0.087 | 0.429 |
|  |  |  | summer | 0.167 | 0.037 – 0.746 |  |  |  |  |
|  |  |  | autumn | 0.304 | 0.040 – 2.294 |  |  |  |  |
|  |  | sex (female) | male | 6.102 | 1.528 – 24.369 | 6.554 | 1 | **0.010** |  |
|  |  | age (< 3 yrs) | ≥ 3 yrs | 0.028 | 0.006 – 0.130 | 20.681 | 1 | **< 0.001** |  |
|  |  | Site (MS) | BB | 1.098 | 0.380 – 3.173 | 0.030 | 1 | 0.863 |  |
|  | 132 | date |  | 1.011 | 1.003 – 1.019 | 8.036 | 1 | **0.005** | 0.444 |
|  |  | date^2^ |  |  |  | 2.976 | 1 | 0.084 |  |
|  |  | sex (female) | male | 4.329 | 1.059 – 17.701 | 4.160 | 1 | **0.041** |  |
|  |  | age (< 3 yrs) | ≥ 3 yrs | 0.033 | 0.007 – 0.161 | 17.816 | 1 | **< 0.001** |  |
|  |  | Site (MS) | BB | 1.417 | 0.466 – 4.307 | 0.379 | 1 | 0.538 |  |
|  | 123 | (breeding) | non-breeding | 4.036 | 1.331 – 12.244 | 6.073 | 1 | **0.014** | 0.464 |
|  |  | sex (female) | male | 4.541 | 1.024 – 20.145 | 3.964 | 1 | **0.046** |  |
|  |  | age (< 3 yrs) | ≥ 3 yrs | 0.027 | 0.005 – 0.152 | 16.832 | 1 | **< 0.001** |  |
|  |  | Site (MS) | BB | 1.242 | 0.417 – 3.705 | 0.152 | 1 | 0.697 |  |
| non-breeding | 44 | season (winter) | summer | 0.517 | 0.022 – 12.346 | 1.884 | 2 | 0.390 | 0.389 |
|  |  |  | autumn | 0.220 | 0.025 – 1.908 |  |  |  |  |
|  |  | sex (female) | male | 9.644 | 1.275 – 72.964 | 4.818 | 1 | **0.028** |  |
|  |  | age (< 3 yrs) | ≥ 3 yrs | 0.046 | 0.005 – 0.380 | 8.140 | 1 | **0.004** |  |
|  |  | Site (MS) | BB | 0.878 | 0.164 – 4.701 | 0.023 | 1 | 0.879 |  |
|  | 44 | date |  | 0.953 | 0.868 – 1.045 | 1.066 | 1 | 0.302 | 0.377 |
|  |  | date^2^ |  |  |  | 0.772 | 1 | 0.380 |  |
|  |  | sex (female) | male | 9.405 | 1.292 – 68.442 | 4.899 | 1 | **0.027** |  |
|  |  | age (< 3 yrs) | ≥ 3 yrs | 0.046 | 0.006 – 0.373 | 8.328 | 1 | **0.004** |  |
|  |  | Site (MS) | BB | 0.554 | 0.061 – 5.065 | 0.274 | 1 | 0.601 |  |
|  | 44 | age (< 3 yrs) | ≥ 3 yrs | 0.148 | 0.034 – 0.642 | 6.512 | 1 | **0.011** | 0.221 |
|  |  | Site (MS) | BB | 1.218 | 0.319 – 4.650 | 0.083 | 1 | 0.773 |  |

Models with quadratic date terms only are shown, as they showed showed better fit (based on AICc and Nagelkerke R^2^) than models with quadratic cubic as well as quadratic date terms. Effect sizes are given as odds ratio (OR), where an OR > 1 indicates that the given category is more likely to be infected than the reference category. Reference categories are shown in parentheses. Sites are Bellbrae (BB) and Meredith/She Oaks (MS).

**Supplementary Table S9:** The effects on BFDV prevalence in cloacal swabs of P. elegans (as reported in Supplementary Table S4), whilst controlling for calendar year of sampling. Subset ‘all’ contains all birds tested regardless of age, sex and breeding status; subset ‘non-breeding’ contains birds that were caught outside the breeding season in walk-in traps. Predictor ‘breeding status’ refers to whether birds were considered ‘non-breeding’ or ‘breeding’ (i.e. were caught during the breeding season in nest boxes). Significant results are shown in bold print. Sample sizes vary due to use of different subsets of birds, and because some sample types could not be obtained from some individuals.

| **subset** | ***n* birds** | **predictor** |  | **OR** | **95% CI for OR** | **Wald χ^2^** | **df** | **p** | **R^2^** |
| --- | --- | --- | --- | --- | --- | --- | --- | --- | --- |
| all | 120 | blood (not infected) | infected | 2.868 | 0.856 – 9.608 | 2.919 | 1 | 0.088 | 0.425 |
|  |  | date |  | 1.010 | 0.993 – 1.027 | 1.424 | 1 | 0.233 |  |
|  |  | date^2^ |  |  |  | 2.423 | 1 | 0.120 |  |
|  |  | date^3^ |  |  |  | 0.350 | 1 | 0.554 |  |
|  |  | sex (female) | male | 1.513 | 0.348 – 6.571 | 0.305 | 1 | 0.581 |  |
|  |  | age (< 3 yrs) | ≥ 3 yrs | 0.075 | 0.015 – 0.373 | 10.036 | 1 | **0.002** |  |
|  |  | year (2018) | 2016 | 250.000 | 0.008 – 7,230,657.990 | 1.686 | 2 | 0.430 |  |
|  |  |  | 2017 | 125.000 | 0.005 – 3,223,726.630 |  |  |  |  |
|  | 120 | date |  | 1.012 | 0.996 – 1.029 | 2.257 | 1 | 0.133 | 0.40 |
|  |  | date^2^ |  |  |  | 1.262 | 1 | 0.226 |  |
|  |  | date^3^ |  |  |  | 0.415 | 1 | 0.519 |  |
|  |  | sex (female) | male | 2.001 | 0.478 – 8.377 | 0.901 | 1 | 0.343 |  |
|  |  | age (< 3 yrs) | ≥ 3 yrs | 0.047 | 0.010 – 0.214 | 15.534 | 1 | **< 0.001** |  |
|  |  | year (2018) | 2016 | 76.923 | 0.006 – 999,000.999 | 1.633 | 2 | 0.442 |  |
|  |  |  | 2017 | 37.037 | 0.003 – 419,815.281 |  |  |  |  |
|  | 114 | (breeding) | non-breeding | 3.955 | 1.145 – 13.668 | 4.724 | 1 | **0.030** | 0.375 |
|  |  | sex (female) | male | 2.401 | 0.585 – 9.845 | 1.479 | 1 | 0.224 |  |
|  |  | age (< 3 yrs) | ≥ 3 yrs | 0.054 | 0.012 – 0.239 | 14.769 | 1 | **< 0.001** |  |
|  |  | year (2018) | 2016 | 35.714 | 2.016 – 500.000 | 5.949 | 2 | 0.051 |  |
|  |  |  | 2017 | 16.667 | 1.387 – 200.000 |  |  |  |  |
| non-breeding | 40 | date |  | 1.210 | 0.934 – 1.566 | 2.087 | 1 | 0.149 | 0.362 |
|  |  | date^2^ |  |  |  | 1.531 | 1 | 0.216 |  |
|  |  | date^3^ |  |  |  | 1.216 | 1 | 0.270 |  |
|  |  | sex (female) | male | 0.629 | 0.093 – 4.231 | 0.228 | 1 | 0.633 |  |
|  |  | age (< 3 yrs) | ≥ 3 yrs | 0.168 | 0.023 – 1.218 | 3.115 | 1 | 0.078 |  |
|  |  | year (2018) | 2017 | 138,966.092 | 1.987E15 – 1.987E15 | 0.985 | 1 | 0.321 |  |
|  | 40 | season |  | Model did  not converge |  |  |  |  |  |
|  |  | sex (female) | male |  |  |  |  |  |  |
|  |  | age (< 3 yrs) | ≥ 3 yrs |  |  |  |  |  |  |
|  |  | year (2018) | 2017 |  |  |  |  |  |  |
|  | 40 | age (< 3 yrs) | ≥ 3 yrs | 0.160 | 0.036 – 0.715 | 5.757 | 1 | **0.016** | 0.257 |
|  |  | year (2018) | 2017 | 10.000 | 0.919 – 111.111 | 3.574 | 1 | 0.059 |  |

For cloacal swabs, models including cubic as well as quadratic date terms are shown, as they showed better fit (based on AICc and Nagelkerke R^2^) than models that included only quadratic date terms. Effect sizes are given as odds ratio (OR), where an OR > 1 indicates that the given category is more likely to be infected than the reference category. Reference categories are shown in parentheses.

**Supplementary Table S10:** The effects on BFDV prevalence in cloacal swabs of P. elegans (as reported in Supplementary Table S4), whilst controlling for site (field site where samples were collected). Subset ‘all’ contains all birds tested regardless of age, sex and breeding status; subset ‘non-breeding’ contains birds that were caught outside the breeding season in walk-in traps. Predictor ‘breeding status’ refers to whether birds were considered ‘non-breeding’ or ‘breeding’ (i.e. were caught during the breeding season in nest boxes). Significant results are shown in bold print. Sample sizes vary due to use of different subsets of birds, and because some sample types could not be obtained from some individuals.

| **subset** | ***n* birds** | **predictor** |  | **OR** | **95% CI for OR** | **Wald χ^2^** | **df** | **p** | **R^2^** |
| --- | --- | --- | --- | --- | --- | --- | --- | --- | --- |
| all | 120 | blood (not infected) | infected | 2.682 | 0.822 – 8.752 | 2.673 | 1 | 0.102 | 0.425 |
|  |  | date |  | 1.016 | 1.005 – 1.028 | 8.321 | 1 | **0.004** |  |
|  |  | date^2^ |  |  |  | 4.509 | 1 | **0.034** |  |
|  |  | date^3^ |  |  |  | 7.551 | 1 | **0.006** |  |
|  |  | sex (female) | male | 1.258 | 0.299 – 5.296 | 0.098 | 1 | 0.755 |  |
|  |  | age (< 3 yrs) | ≥ 3 yrs | 0.101 | 0.023 – 0.456 | 8.916 | 1 | **0.003** |  |
|  |  | Site (MS) | BB | 2.121 | 0.704 – 6.392 | 1.784 | 1 | 0.182 |  |
|  | 120 | date |  | 1.017 | 1.006 – 1.028 | 9.021 | 1 | **0.003** | 0.403 |
|  |  | date^2^ |  |  |  | 3.497 | 1 | 0.61 |  |
|  |  | date^3^ |  |  |  | 6.693 | 1 | **0.010** |  |
|  |  | sex (female) | male | 1.691 | 0.411 – 6.957 | 0.530 | 1 | 0.467 |  |
|  |  | age (< 3 yrs) | ≥ 3 yrs | 0.062 | 0.015 – 0.261 | 14.425 | 1 | **< 0.001** |  |
|  |  | Site (MS) | BB | 2.168 | 0.732 – 6.421 | 1.951 | 1 | 0.162 |  |
|  | 114 | (breeding) | non-breeding | 2.172 | 0.812 – 5.813 | 2.386 | 1 | 0.122 | 0.320 |
|  |  | sex (female) | male | 1.591 | 0.474 – 5.346 | 0.564 | 1 | 0.452 |  |
|  |  | age (< 3 yrs) | ≥ 3 yrs | 0.101 | 0.029 – 0.355 | 12.747 | 1 | **< 0.001** |  |
|  |  | Site (MS) | BB | 1.768 | 0.697 – 4.482 | 1.442 | 1 | 0.230 |  |
| non-breeding | 40 | date |  | 1.108 | 0.919 – 1.335 | 1.152 | 1 | 0.283 | 0.311 |
|  |  | date^2^ |  |  |  | 0.552 | 1 | 0.458 |  |
|  |  | date^3^ |  |  |  | 0.183 | 1 | 0.668 |  |
|  |  | sex (female) | male | 0.781 | 0.122 – 4.990 | 0.068 | 1 | 0.794 |  |
|  |  | age (< 3 yrs) | ≥ 3 yrs | 0.142 | 0.020 – 0.985 | 3.900 | 1 | **0.048** |  |
|  |  | Site (MS) | BB | 0.961 | 0.082 – 11.322 | 0.001 | 1 | 0.975 |  |
|  | 40 | season (winter) | summer | 0.188 | 0.011 – 3.155 | 1.367 | 2 | 0.505 | 0.177 |
|  |  |  | autumn | 0.620 | 0.097 – 3.968 |  |  |  |  |
|  |  | sex (female) | male | 0.897 | 0.181 – 4.441 | 0.018 | 1 | 0.894 |  |
|  |  | age (< 3 yrs) | ≥ 3 yrs | 0.231 | 0.043 – 1.251 | 2.888 | 1 | 0.089 |  |
|  |  | Site (MS) | BB | 0.548 | 0.099 – 3.041 | 0.473 | 1 | 0.491 |  |
|  | 40 | age (< 3 yrs) | ≥ 3 yrs | 0.246 | 0.060 – 1.011 | 3.781 | 1 | 0.052 | 0.130 |
|  |  | Site (MS) | BB | 0.864 | 0.219 – 3.419 | 0.043 | 1 | 0.835 |  |

For cloacal swabs, models including cubic as well as quadratic date terms are shown, as these showed better fit (based on AICc and Nagelkerke R^2^) than models that included only quadratic date terms. Effect sizes are given as odds ratio (OR), where an OR > 1 indicates that the given category is more likely to be infected than the reference category. Reference categories are shown in parentheses. Sites are Bellbrae (BB) and Meredith/She Oaks (MS).

**Supplementary Table S11:** Example comparison of three different Julian day factors, as conducted during exploratory data analysis. The three different date factors gave almost identical results and have a similar model fit, as indicated by the Nagelkerke R^2^ and AICc values. ‘January date’ is Julian day with the 1^st^ January as day 1, ‘February date’ is with the 1^st^ February as day 1 (this is the measure of Julian day which we use for analyses elsewhere in this study) and ‘September date’ is with the 1^st^ September as day 1. All models were run with the data set ‘all’ (see Supplementary Tables S3 – S10), which contains all birds tested, regardless of their age, sex and breeding status. Sample sizes vary because some sample types could not be obtained from some individuals.

| **sample type** | ***n* birds** | **predictor** |  | **OR** | **95% CI for OR** | **Wald χ^2^** | **df** | **p** | **R^2^** | **AICc** |
| --- | --- | --- | --- | --- | --- | --- | --- | --- | --- | --- |
| blood samples | 132 | January date |  | 1.011 | 1.003 – 1.018 | 7.836 | 1 | **0.005** | 0.441 | 98.932 |
|  |  | January date^2^ |  |  |  | 3.993 | 1 | **0.046** |  |  |
|  |  | sex (female) | male | 4.493 | 1.098 – 18.380 | 4.370 | 1 | **0.037** |  |  |
|  |  | age (< 3 yrs) | ≥ 3 yrs | 0.032 | 0.006 – 0.154 | 18.305 | 1 | **< 0.001** |  |  |
|  |  | February date |  | 1.011 | 1.003 – 1.019 | 7.871 | 1 | **0.005** | 0.441 | 98.932 |
|  |  | February date^2^ |  |  |  | 3.993 | 1 | **0.046** |  |  |
|  |  | sex (female) | male | 4.493 | 1.098 – 18.380 | 4.370 | 1 | **0.037** |  |  |
|  |  | age (< 3 yrs) | ≥ 3 yrs | 0.032 | 0.006 – 0.154 | 18.305 | 1 | **< 0.001** |  |  |
|  |  | September date |  | 1.001 | 0.995 – 1.009 | 0.174 | 1 | 0.676 | 0.438 | 94.881 |
|  |  | September date^2^ |  |  |  | 4.270 | 1 | **0.039** |  |  |
|  |  | sex (female) | male | 6.186 | 1.580 – 24.215 | 6.851 | 1 | **0.009** |  |  |
|  |  | age (< 3 yrs) | ≥ 3 yrs | 0.027 | 0.006 – 0.126 | 21.141 | 1 | **< 0.001** |  |  |
| cloacal swabs | 120 | January date |  | 1.005 | 0.999 – 1.012 | 2.312 | 1 | 0.128 | 0.332 | 110.191 |
|  |  | January date^2^ |  |  |  | 3.242 | 1 | 0.072 |  |  |
|  |  | sex (female) | male | 1.999 | 0.545 – 7.331 | 1.091 | 1 | 0.296 |  |  |
|  |  | age (< 3 yrs) | ≥ 3 yrs | 0.072 | 0.019 – 0.276 | 14.745 | 1 | **< 0.001** |  |  |
|  |  | February date |  | 1.005 | 0.999 – 1.012 | 2.379 | 1 | 0.123 | 0.332 | 110.191 |
|  |  | February date^2^ |  |  |  | 3.242 | 1 | 0.072 |  |  |
|  |  | sex (female) | male | 1.999 | 0.545 – 7.331 | 1.091 | 1 | 0.296 |  |  |
|  |  | age (< 3 yrs) | ≥ 3 yrs | 0.072 | 0.019 – 0.276 | 14.745 | 1 | **< 0.001** |  |  |
|  |  | September date |  | 0.996 | 0.988 – 1.003 | 1.187 | 1 | 0.276 | 0.326 | 108.071 |
|  |  | September date^2^ |  |  |  | 0.012 | 1 | 0.911 |  |  |
|  |  | sex (female) | male | 1.477 | 0.440 – 4.955 | 0.399 | 1 | 0.528 |  |  |
|  |  | age (< 3 yrs) | ≥ 3 yrs | 0.096 | 0.028 – 0.327 | 14.072 | 1 | **< 0.001** |  |  |
